# TNF-α-dependent neuronal necroptosis regulated in Alzheimer's disease by coordination of RIPK1-p62 complex with autophagic UVRAG

**DOI:** 10.7150/thno.62376

**Published:** 2021-09-13

**Authors:** Chong Xu, Jialin Wu, Yiqun Wu, Zhichu Ren, Yuyuan Yao, Guobing Chen, Evandro F. Fang, Ji Heon Noh, Yong U. Liu, Libin Wei, Xijing Chen, Jian Sima

**Affiliations:** 1 Laboratory of Aging Neuroscience and Neuropharmacology, School of Basic Medicine and Clinical Pharmacy, China Pharmaceutical University, Nanjing, 210009, China.; 2 Institute of Geriatric Immunology, School of Medicine, Jinan University, Guangzhou, 510632, China.; 3 Department of Clinical Molecular Biology, University of Oslo and Akershus University Hospital, 1478 Lørenskog, Norway.; 4 The Norwegian Centre on Healthy Ageing (NO-Age), Oslo, Norway.; 5 Department of Biochemistry, Chungnam National University, Daehak-ro 99, Yuseong-gu, Daejeon.; 6 Laboratory for Neuroscience in Health and Disease, Guangzhou First People's Hospital, South China University of Technology, Guangzhou, 510180, China.; 7 Jiangsu Key Laboratory of Carcinogenesis and Intervention, China Pharmaceutical University, Nanjing, 210009, China.; 8 Clinical Pharmacokinetics Laboratory, School of Basic Medicine and Clinical Pharmacy, China Pharmaceutical University, Nanjing, 211198, China.

**Keywords:** Alzheimer's disease, neuronal necroptosis, TNF-α/TNFR1 signaling, RIPK1/RIPK3/MLKL cascade, autophagic flux, p62, UVRAG

## Abstract

**Background:** Neuronal death is a major hallmark of Alzheimer's disease (AD). Necroptosis, as a programmed necrotic process, is activated in AD. However, what signals and factors initiate necroptosis in AD is largely unknown.

**Methods:** We examined the expression levels of critical molecules in necroptotic signaling pathway by immunohistochemistry (IHC) staining and immunoblotting using brain tissues from AD patients and AD mouse models of APP/PS1 and 5×FAD. We performed brain stereotaxic injection with recombinant TNF-α, anti-TNFR1 neutralizing antibody or AAV-mediated gene expression and knockdown in APP/PS1 mice. For *in vitro* studies, we used TNF-α combined with zVAD-fmk and Smac mimetic to establish neuronal necroptosis models and utilized pharmacological or molecular biological approaches to study the signaling pathways.

**Results:** We find that activated neuronal necroptosis is dependent on upstream TNF-α/TNFR1 signaling in both neuronal cell cultures and AD mouse models. Upon TNF-α stimulation, accumulated p62 recruits RIPK1 and induces its self-oligomerization, and activates downstream RIPK1/RIPK3/MLKL cascade, leading to neuronal necroptosis. Ectopic accumulation of p62 is caused by impaired autophagy flux, which is mediated by UVRAG downregulation during the TNF-α-promoted necroptosis. Notably, UVRAG overexpression inhibits neuronal necroptosis in cell and mouse models of AD.

**Conclusions:** We identify a finely controlled regulation of neuronal necroptosis in AD by coordinated TNF-α signaling, RIPK1/3 activity and autophagy machinery. Strategies that could fine-tune necroptosis and autophagy may bring in promising therapeutics for AD.

## Introduction

Alzheimer's disease (AD) is a progressive neurodegenerative disorder characterized by clinical symptoms of memory loss, personality changes, and general cognitive decline 1, 2. The neuropathological alterations of AD brain include Amyloid-β (Aβ) deposit, aggregates of hyperphosphorylated/misfolded tau, neuroinflammation, and neuronal loss 3, 4. Despite continuing debates about hypotheses for the cause(s) of AD, Aβ-induced neuronal death and brain dystrophy has emerged as the most extensively known theory 5. In the past decades, evidence has shown that extracellular Aβ plaques are directly toxic to adjacent neurons 6, and soluble or aggregated Aβ can also induce inflammatory processes and secondary cell death 7. Among the various forms of cell death pathways, while it is still debating the role of neuronal apoptosis in AD, increased necrosis and microglial phagocytosis are likely playing important roles in neuronal death in AD 8-10. Recent findings have also implicated necroptosis, a programmed cell necrosis, in the aetiology of neuronal loss of AD 11-13. However, the mechanism initiating necroptosis in AD is not known.

Another process that may be involved in AD pathology is the age- or genetic-dependent compromisation of autophagy 14. Autophagy is a central cellular process that cleaves molecules and subcellular elements, including nucleic acids, proteins, lipids and organelles, via lysosome-mediated degradation to promote homeostasis, differentiation, development and survival 15. Recent studies also suggest its function in modulating the switching between apoptosis and necroptosis 16. Interestingly, a considerable amount of investigation has found that impaired autophagy contributes to the accumulation of misfolded protein aggregates, which is featured by most neurodegenerative diseases (NDs), including AD 17. However, very little is currently known about the possible interplay between autophagy pathway and neuronal necroptosis in AD pathogenesis.

We have turned to investigation of possible action of a candidate critical proinflammatory cytokine in these processes: tumor necrosis factor-α (TNF-α). It is already known to be involved in neuroinflammation and to contribute to AD pathology 18, 19. TNF-α is elevated in the cerebrospinal fluid (CSF) and plasma in aging, mild cognitive impairment (MCI), and AD patients, and MCI patients with a higher level of TNF-α are much more likely to progress to full AD 20. Data from animal models further reveal that TNF-α-induced inflammation may have a detrimental effect on neuronal death, and chronic release of TNF-α during AD progression is likely through the activation of microglia, astrocytes and neurons stimulated by aggregated Aβ 7, 21. Currently, strategies targeting TNF-α inhibition have demonstrated some reduction of brain pathology and alleviation of cognitive decline in both rodent models and in AD patients 19, 21.

TNF-α acts through a classical signaling pathway initiated by binding to its receptor TNFR1. TRADD, TRAF2/5, RIPK1, cIAP1/2 and other subunits are then recruited to form complex I, which in turn leads to ubiquitination of RIPK1 and thus to NF-κB activation 22. When NF-κB activation is inhibited, e.g., by blocking cIAP1/2, caspase-8 can be activated, which induces RIPK1 cleavage and leads to cell apoptosis. Elimination or inhibition of caspase-8 prompts RIPK1 activation through deubiquitination and leads to its interaction with FADD, TRADD, and RIPK3, to form complex II. Complex II can phosphorylate the pseudokinase MLKL and ultimately promotes necrotic cell membrane disruption and cell death, which is also termed necroptosis 23, 24. However, whether elevated TNF-α in AD can induce neuronal necroptosis has remained conjectural.

We now report that elevated TNF-α signaling can indeed induce the activation of neuronal necroptosis in AD. TNF-α-triggered neuronal necroptosis is further modulated by impaired autophagic flux, in which a key regulator UVRAG tightly regulates signaling transduction. These findings thus implicate crosstalk between neuronal necroptosis and autophagy machinery in AD pathology, and may exemplify a more general paradigm for cell death regulation in other NDs.

## Results

### TNF-α signaling promotes neuronal necroptosis in AD mice

**Figure 1:**
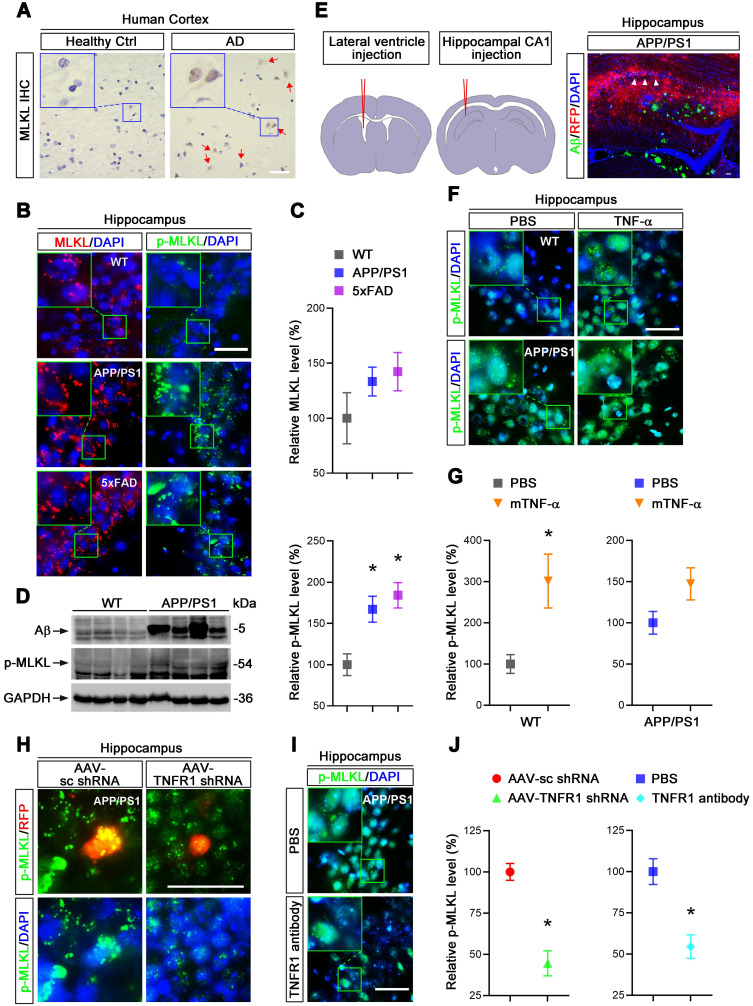
** TNF-α/TNFR1-dependent neuronal necroptosis is activated in AD models. (A)** DAB staining immunohistochemistry (IHC) shows the MLKL levels in the cortexes from AD patients and healthy controls. Nuclei were counterstained with hematoxylin. Arrows indicate positive IHC staining. **(B)** Immunofluorescence (IF) images show the levels of MLKL (red) or p-MLKL (green) in the hippocampal CA1 regions from WT, APP/PS1 and 5×FAD mice respectively. Nuclei were counterstained with DAPI. **(C)** Quantitation of MLKL and p-MLKL levels in (B). The protein levels in WT were normalized as 100%. **(D)** Immunoblotting shows the protein levels of Aβ and p-MLKL in hippocampus from WT and APP/PS1 mice. **(E)** Schematic overview shows lateral ventricle injection of murine TNF-α (left) and intrahippocampal CA1 injection of anti-TNFR1 neutralizing antibody or AAV particles (middle). Image shows Aβ IF staining and RFP in AAV-transduced cells in APP/PS1 mice (right). Arrowheads, CA1 region. **(F)** IF images show p-MLKL (green) levels in WT or APP/PS1 with mTNF-α or PBS injection. **(G)** Quantitation of p-MLKL levels in (F). **(H)** IF images show the levels of p-MLKL (green) in APP/PS1 mice injected with AAV-scramble control (sc) shRNA-RFP or AAV-TNFR1 shRNA-RFP. **(I)** IF images show the p-MLKL (green) levels in WT or APP/PS1 with or without anti-TNFR1 neutralizing antibody injection. **(J)** Quantitation of p-MLKL levels in (H) and (I). Scale bar, 25 μm. Error bars indicate mean ± SEM from at least 20 RFP-labeled cells, n=3~5 mice per group; ^*^p < 0.05, one-way ANOVA test.

We hypothesized that elevated TNF-α signaling in AD brain may lead to neuronal necroptosis. To test this hypothesis, we first examined the levels of MLKL and phosphorylated-MLKL (p-MLKL), a marker of cell necroptosis, in the brains of AD patients. Immunohistochemical (IHC) staining demonstrated upregulation of both MLKL and p-MLKL in samples of cerebral cortex from AD patients at Braak VI stage compared to age-matched healthy controls (Ctrl) (Figure 1A, Figure S1). We then checked the levels of total MLKL and phosphorylated-MLKL (p-MLKL) in 10-month old APP/PS1 and 5×FAD transgenic AD mouse models. IHC images of hippocampal CA1 regions showed that compared to wild type (WT) littermate controls, p-MLKL levels were greatly increased in either APP/PS1 or 5×FAD mice, although total MLKL levels were only mildly augmented (Figure 1B & C). We noted that 5×FAD showed stronger activation of MLKL compared to APP/PS1 mice, possibly due to their more severe AD pathology 25. In addition to the IHC data, immunoblotting of whole brain lysate also showed a higher p-MLKL level in 10-month old APP/PS1 mice compared to WT littermates (Figure 1D). These data indicate that in line with previous findings 11, necroptosis is indeed activated in AD.

To assess whether TNF-α signaling is involved in neuronal necroptosis in the hippocampus, we carried out lateral ventricle injection of murine TNF-α as described 26 (Figure 1E left). Notably, 5 μg TNF-α compared to PBS control injection induced p-MLKL levels ~3 fold in the cells of CA1 pyramidal layers (Figure 1F & G), suggesting that a high level of TNF-α is sufficient to activate necroptosis in mouse hippocampus. In addition, TNF-α injection into APP/PS1 hippocampus only slightly increased the levels of p-MLKL (~1.47 fold), perhaps because the level of p-MLKL activated in APP/PS1 mice was already so high (Figure 1F & G). We also found that TNF-α injection in 10-month old WT mice induced ~13.75% neuronal loss compared to PBS injection (Figure S2), consistent with the necroptosis activation induced by TNF-α.

To further study the function of TNF-α signaling in necroptosis, we used adeno-associated virus (AAV) vector-mediated small hairpin RNA (shRNA) to knockdown (KD) TNFR1 (Figure S4A), a TNF-α receptor mediating cell death signaling. Assessed by IHC and immunoblotting at least 70% reduction of TNFR1 efficiency were seen (Figure S3A, C). Using intrahippocampal injection (Figure 1E middle and right), we transduced CA1 cells with AAV particles expressing a TNFR1 shRNA or a scrambled control (sc) shRNA in 10-month old APP/PS1 mice. After 3 weeks, p-MLKL levels were then examined by IHC. Data showed that TNFR1 shRNA reduced p-MLKL levels by 55.5% compared to sc shRNA (Figure 1H, J left). By injection of an anti-TNFR1 neutralizing antibody that can block TNF-α signaling 27, we also observed an average 45.5% reduction of p-MLKL levels in CA1 neurons (Figure 1I, J right).

In sum, we infer TNF-α/TNFR1 signaling is indispensable for the neuronal necroptosis activated in AD patients and AD mouse models.

### TNF-α can trigger neuronal necroptosis in cell culture

**Figure 2:**
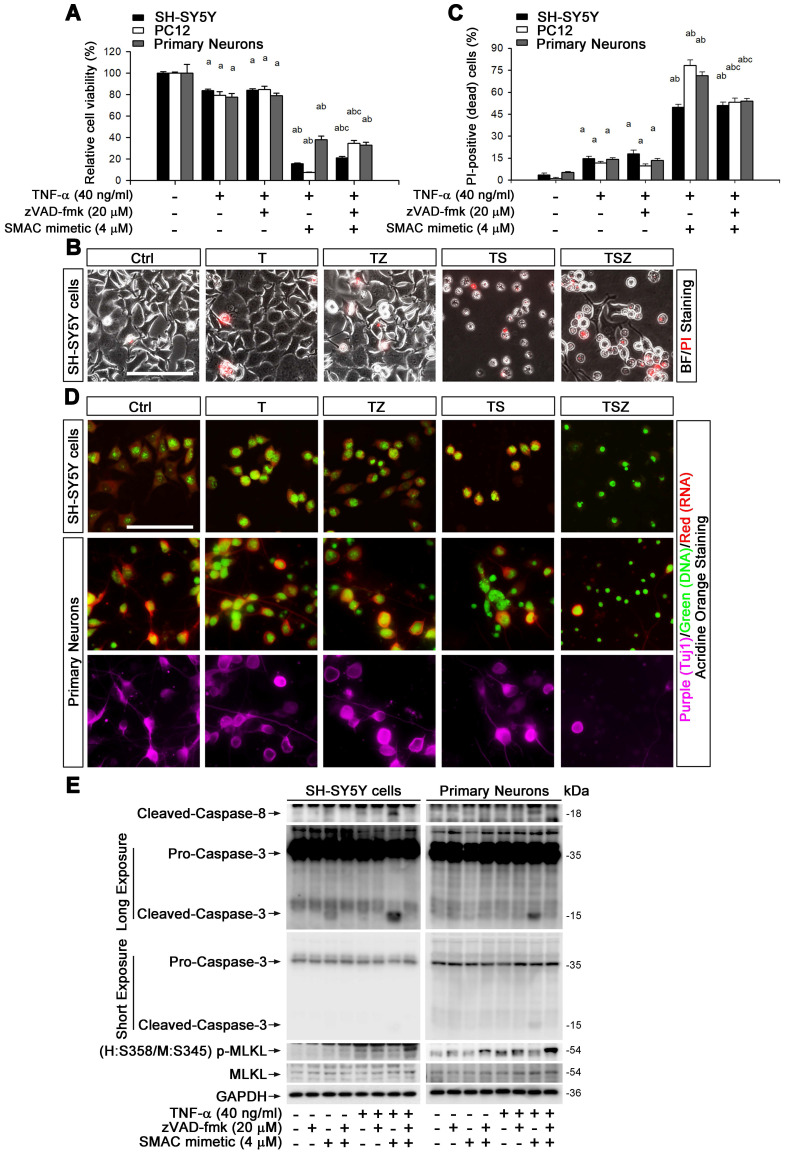
** TNF-α induces neuronal necroptosis in cell cultures. (A)** Quantitation of cell viability from neuronal cell cultures with or without indicated TNF-α (T), zVAD (Z), and SMAC mimetic (S) treatment. **(B)** Images of propidium iodide (PI) staining (red) in SH-SY5Y cells after TSZ treatment. BF, bright field. **(C)** Quantitation of PI-positive cells from SH-SY5Y, PC12 and primary neurons after indicated treatments. **(D)** Images of acridine orange (AO) staining of nuclei (green) and cytoplasm (red) in TSZ treated SH-SY5Y cells (upper panels) and in primary neurons (lower panels). Tuj1 (purple) as a neuronal marker. **(E)** Immunoblotting shows levels of Caspase 3, Caspase 8, p-MLKL, and MLKL in SH-SY5Y cells (left panels) and in primary neurons (right panels) with TSZ treatments. GADPH as a loading control. Scale bar, 25 μm. ^a^*p* < 0.05, difference with control group; ^b^*p* < 0.05, difference with TNF-α treated group; ^c^*p* < 0.05, difference with TS treated group. Data represent mean ± SEM, n=3~5 independent experiments; analysis was performed by one-way ANOVA test.

To explore the molecular mechanism of TNF-α-mediated neuronal necroptosis, we performed cell culture experiments using various neuronal cell types including SH-SY5Y, PC-12 and primary cortical neurons. We examined the effects of TNF-α on cell viability in all above cell types. The pan-caspase inhibitor zVAD-fmk (zVAD, Z) and cIAPs inhibitor Smac mimetic (S), two inducers of cell necroptosis 28, were also utilized. We found that 40 ng/ml TNF-α alone (T) induced a loss of cell viability in all three cell types, with a percentage of 16.4% (SH-SY5Y), 21.5% (PC12), and 22.4% (primary neurons), but zVAD did not fully block cell death (Figure 2A, Figure S5A). We further observed that Smac mimetic remarkably increased TNF-α-induced cell death, which could only be partially inhibited by zVAD, with a percentage of 5.5% in SH-SY5Y or 27% in PC12. Moreover, zVAD even provoked cell death in primary neurons with a percentage of 4.9% (Figure 2A). These data imply that TNF-α or TSZ treatment can induce type(s) of cell death besides common apoptosis.

Propidium iodide (PI) staining and cell morphology images showed that TNF-α, especially combined with Smac mimetic and zVAD (TSZ) treatment, indeed triggered the characteristic features of necrosis/necroptosis 29, including PI-positive labeling and cell swelling (Figure 2B). Quantitation indicated that TSZ increased cell necrosis with a percentage of 51% in SH-SY5Y, 53.2% in PC12 or 54% in primary neurons; TNF-α treatment also slightly increased necrosis with a percentage of 11.7% in PC12, 14.7% in SH-SY5Y, and 14% in primary neurons (Figure 2C).

To confirm the involvement of necroptosis in TNF-α-induced cell death, we used Acridine Orange (AO) staining, a unique technique used to distinguish necroptosis from apoptosis 29. After staining, cultured SH-SY5Y cells as well as primary neurons showed flattened morphology with intact cytoplasm (red fluorescence) and nuclei (green fluorescence) (Figure 2D). TNF-α treatment induced cell contraction and nuclear condensation, while TS triggered features of apoptotic cells, with nuclear fragmentation. Particularly, zVAD yielded necrotic cell morphology in TS treated cells with PI-positive nuclei, loss of plasma membrane integrity, and translucent cytosol. zVAD also slightly induced cell necrosis in TNF-α treated cells (Figure 2B-D). To verify that TNF-α-induced cell necrosis is indeed necroptosis, we examined the levels of the specific marker p-MLKL with different combination treatment of T/S/Z. We found that TNF-α or Smac mimetic alone both mildly activated caspase-8 and caspase-3 in SH-SY5Y cells but not in primary neurons, and the activation was inhibited by zVAD (Figure 2E). Interestingly, TS powerfully activated caspase-8 and caspase-3, an action entirely blocked by zVAD (Figure 2E). In addition to the apoptotic caspase activation, TNF-α or TZ treatment indeed upregulated p-MLKL, while TSZ treatment had a sharp effect on p-MLKL (Figure 2E, Figure S5B).

Taken together, our data show that TNF-α alone can mildly activate neuronal necroptosis; but combined with Smac mimetic and zVAD (TSZ), it triggers strong activation of neuronal necroptosis.

### TNF-α-induced neuronal necroptosis is dependent on RIPK1 action

**Figure 3:**
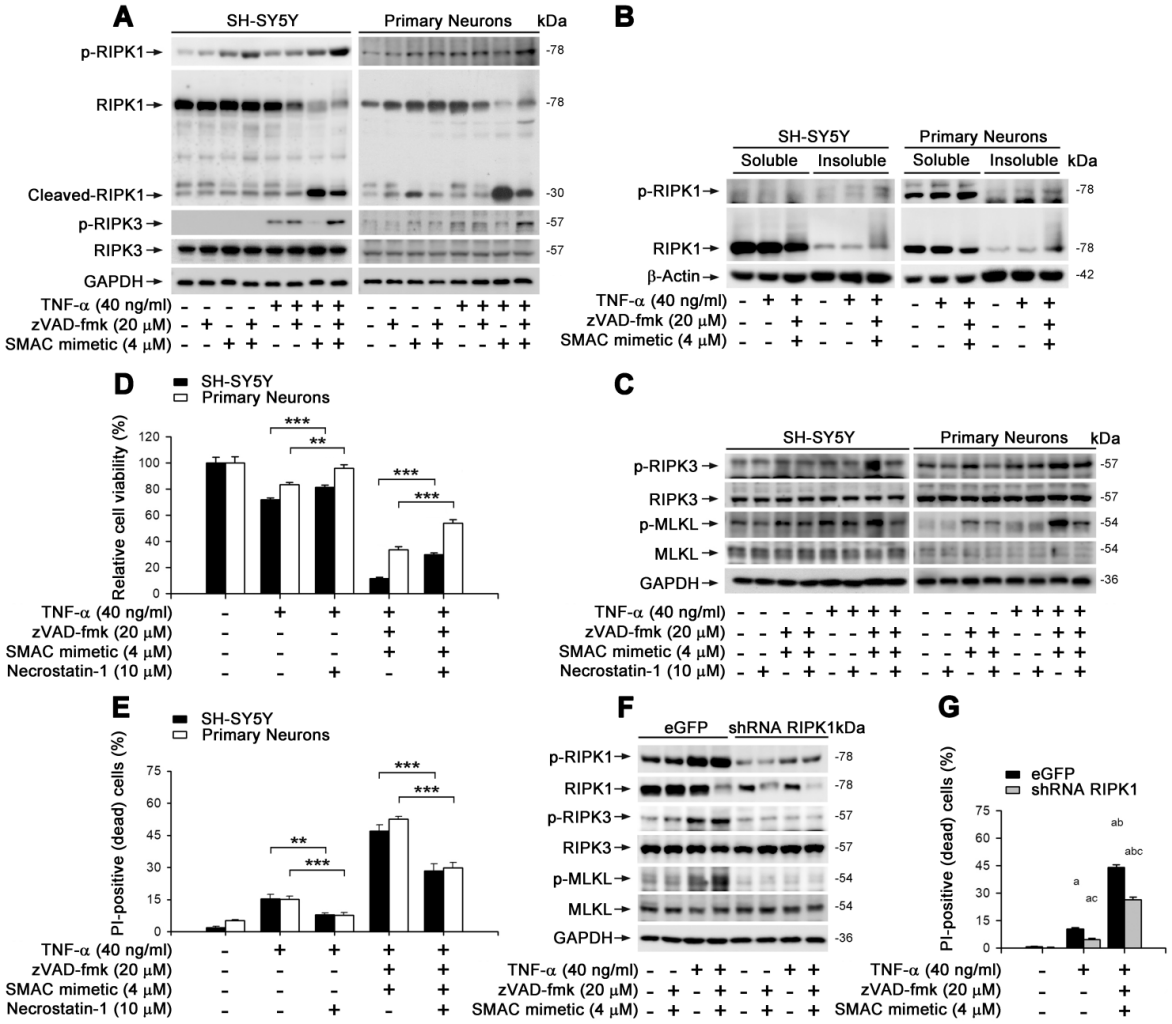
** TNF-α induces neuronal necroptosis is RIPK1-dependent. (A)** Immunoblotting of cell lysates from the SH-SY5Y cells (left panels) or primary neurons (right panels) probed with the indicated antibodies. **(B)** Immunoblotting of soluble and insoluble proteins (see methods) from SH-SY5Y cells (left panels) or primary neurons (right panels) probed with the indicated antibodies. β-Actin as a loading control. **(C)** Immunoblotting of cell lysates from the SH-SY5Y cells (left panels) or primary neurons (right panels) with or without TSZ and Necrostain-1 treatments. **(D)** Quantitation of cell viability from SH-SY5Y cells and primary neurons with or without indicated treatments. **(E)** Quantitation of PI-positive cells from cell cultures with indicated treatments as in (D). **(F)** Immunoblotting of the SH-SY5Y cells transduced with lentiviral particles encoding eGFP (as control) or RIPK1 shRNA. **(G)** Quantitation of PI-positive cells from SH-SY5Y cells expressed eGFP or RIPK1 shRNA with or without indicated treatments. ^a^*p* < 0.05, difference with control group; ^b^*p* < 0.05, difference with TNF-α treated group; ^c^*p* < 0.05, difference with TSZ treated group; ^d^*p* < 0.05, difference with eGFP group. Data represent mean ± SEM, n=3~5 independent experiments; analysis was performed by one-way ANOVA test.

We aimed to clarify whether TNF-α-induced neuronal necroptosis is dependent on RIPK1, a known crucial control center in regulating TNF-α-induced NF-κB signaling pathway as well as apoptosis and necroptosis 23. In cultured SH-SY5Y and primary neurons, similar to MLKL activation (Figure 2E), TNF-α alone lightly induced the phosphorylation of RIPK1 on Ser166 -used as a marker of RIPK1 activation (Figure 3A). TNF-α also increased the phosphorylation of RIPK3, a RIPK1 binding partner, on its Ser227 (human) or Thr231/Ser232 (mouse) site. Smac mimetic, however, markedly repressed RIPK1/3 phosphorylation, while TSZ strongly activated RIPK1 and RIPK3 (Figure 3A). Of note, we observed that zVAD effectively prevented TS induced RIPK1 cleavage (Figure 3A), suggesting a possible promotion of RIPK1 oligomerization and aggregation, additional features of RIPK1-dependent necroptosis 11, 30. As shown in Figure 3B, TNF-α alone or TSZ did not change the RIPK1 levels in the soluble Triton X-100 fractions, but strongly elevated RIPK1 levels in the insoluble urea fractions, indicating an increased RIPK1 aggregation by TSZ treatment. Using an RIPK1 inhibitor Necrostain-1 (Nec-1), we found that Nec-1 effectively suppressed the activation of RIPK3 and MLKL, as well as subsequent cell necroptosis induced by TNF-α or TSZ (Figure 3C-E). Furthermore, RIPK1 reduction by lentiviral vector mediated shRNA distinctly blocked phosphorylation of RIPK1/3 and MLKL, and thereby also blocked cell necroptosis (Figure 3F & G, Figure S4B). Thus, our data suggest that TNF-α-induced neuronal necroptosis is dependent on the activation of an RIPK1/RIPK3/MLKL cascade.

### p62 mediates TNF-α-induced necroptosis via its interaction with RIPK1

**Figure 4:**
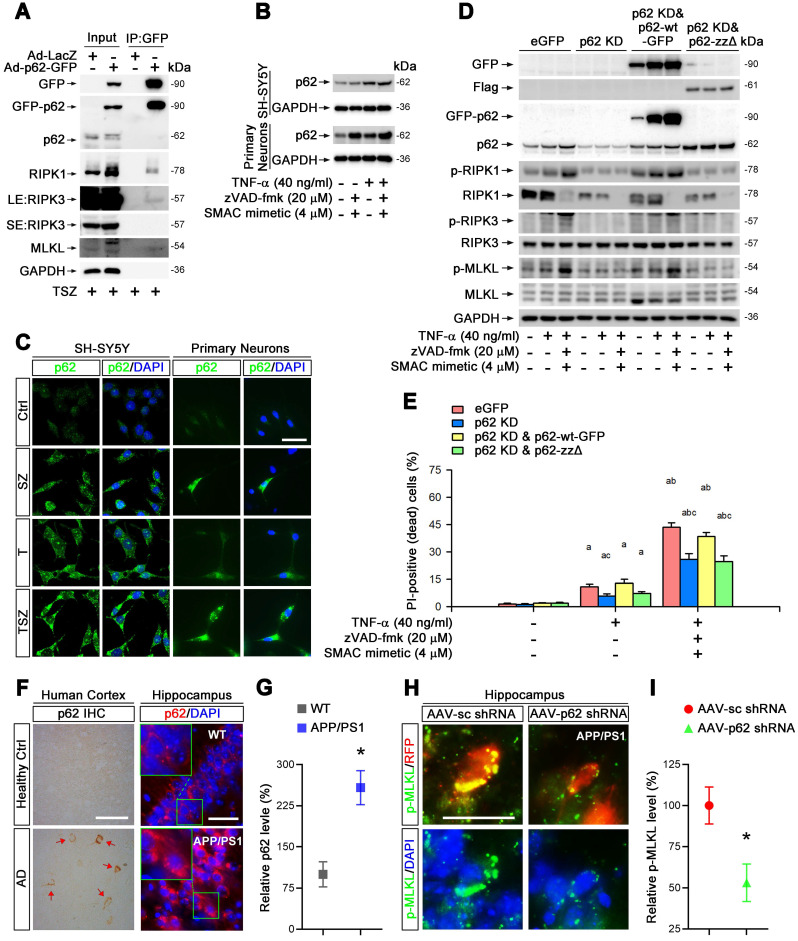
** p62 mediates TNF-α-induced necroptosis via interaction with RIPK1. (A)** GFP immunoprecipitation (IP) with total lysates of SH-SY5Y cells was followed by immunoblotting with the indicated antibodies. **(B)** Immunoblotting shows levels of p62 with indicated TSZ treatments in SH-SY5Y cells (upper panels) or primary neurons (lower panels). GADPH as a loading control. **(C)** IF images show the levels of p62 (green) in SH-SY5Y cells (left panels) or primary neurons (right panels) with indicated treatments. **(D)** Immunoblotting of cell lysates from the SH-SY5Y cells transduced with lentiviral particles expressing eGFP (Ctrl), p62 shRNA (p62 KD), p62 shRNA combined with RNAi-resistant wild type p62 (p62 KD & p62-wt) or p62 shRNA combined with RNAi-resistant zz domain-deleted p62 (p62 KD & p62-zzΔ) using the indicated antibodies. **(E)** Quantitation of PI-positive cells from the SH-SY5Y cells with indicated vector expression and TSZ treatments in (D). **(F)** DAB staining IHC (left) show the p62 levels in the cortical tissues from AD patients and healthy controls. Arrows indicate positive DAB staining. IF (right) show the levels of p62 (red) in the hippocampal CA1 from WT and APP/PS1 mice. Nuclei were counterstained with DAPI. **(G)** Quantitation of p62 levels in (F, right panels). The protein levels in WT were normalized as 100%. **(H)** IF images show the levels of p-MLKL (green) in APP/PS1 injected with AAV particles expressing sc shRNA-RFP (left panels) or p62 shRNA-RFP (right panels). **(I)** Quantitation of p-MLKL levels from RFP-labeled cells in (H). Scale bar, 25 μm. Error bars indicate mean ± SEM from at least 20 RFP-labeled cells, n=3~5 mice per group, or 3~5 independent cell culture experiments. ^*^p < 0.05, difference with WT mice group or sc shRNA injection group; ^a^*p* < 0.05, difference with control group; ^b^*p* < 0.05, difference with TNF-α treated group; ^c^*p* < 0.05, difference with eGFP group; analysis was performed by one-way ANOVA test.

Previous studies have found that RIPK1 interacts directly with p62/SQSTM1, a molecule responsible for the clearance of aggregated proteins 31. We inferred that p62 might participate in RIPK1-dependent neuronal necroptosis in AD. To test this notion, we first examined the protein complex formation of RIPK1, RIPK3 and p62 in neuronal necroptotic cells. Immunoprecipitation data showed that p62 indeed complexed with RIPK1, RIPK3 and MLKL when cells were treated with TSZ (Figure 4A). Endogenous p62 was also upregulated in both SH-SY5Y cells and primary neurons with TNF-α or TSZ treatment (Figure 4B & C).

To evaluate the function of p62 in necroptosis, we transduced SH-SY5Y cells with recombinant adenoviral particles encoding GFP-tagged p62 (Ad-p62-GFP) or encoding LacZ as a control (Ad-LacZ) (Figure S4C). Interestingly, overexpression of p62-GFP evidently upregulated the total protein levels of RIPK1, increased the phosphorylation levels of RIPK1 as well as MLKL, and augmented the percentage of neuronal necroptosis (Figure S6A & B). Consistently, p62 shRNA transduced into cells with lentiviral particles reversed the effect of p62 overexpression (Figure S4B, S6C & D). Furthermore, necroptosis was restored by expression of p62-GFP (shRNA-resistant) in p62 shRNA transfected cells (Figure 4D & E), implying a critical role of p62 in TNF-α-induced neuronal necroptosis.

The ZZ domain (amino acid 122-167) of p62 is the binding domain with RIPK1 32. Here, a ZZ domain deleted p62 (p62-ZZΔ, 3×Flag-tagged) expressing plasmid (Figure S4B) was used to determine whether necroptosis elevation by p62 expression is due to its interaction with RIPK1. Data showed that the expression of p62-ZZΔ in p62 KD cells failed to recover the activation of RIPK1/RIPK3/MLKL pathway and subsequent necroptosis (Figure 4D & E). We conclude that p62 promotes neuronal necroptosis by complexing with RIPK1 and activating MLKL.

To test the possible promotion role of p62 for neuronal necroptosis in AD, we then assessed p62 levels in the brains of AD patients. IHC staining showed higher p62 levels in samples of cerebral cortex from AD patients compared to healthy controls (Figure 4F). Similarly, IHC images of hippocampal CA1 regions also showed remarkable upregulation of p62 in APP/PS1 mice compared to WT littermates (Figure 4F & G). Next, we used AAV vector-mediated shRNA to knock down p62 (Figure S3B, D, S4A) and transduced cells in CA1 regions with AAV particles in APP/PS1 mice by intrahippocampal injection. After three weeks, IHC of p62 and p-MLKL was performed. Our data indicated that p62 shRNA diminished p-MLKL levels by 47% compared to sc shRNA (Figure 4H & I).

Overall, our results demonstrate that p62 elevates neuronal necroptosis by interacting with RIPK1 and promoting phosphorylation of RIPK1/RIPK3/MLKL cascades, both in TNF-α-treated neuronal cultures and in AD mouse brain.

### Impaired autophagic flux regulates neuronal necroptosis

**Figure 5:**
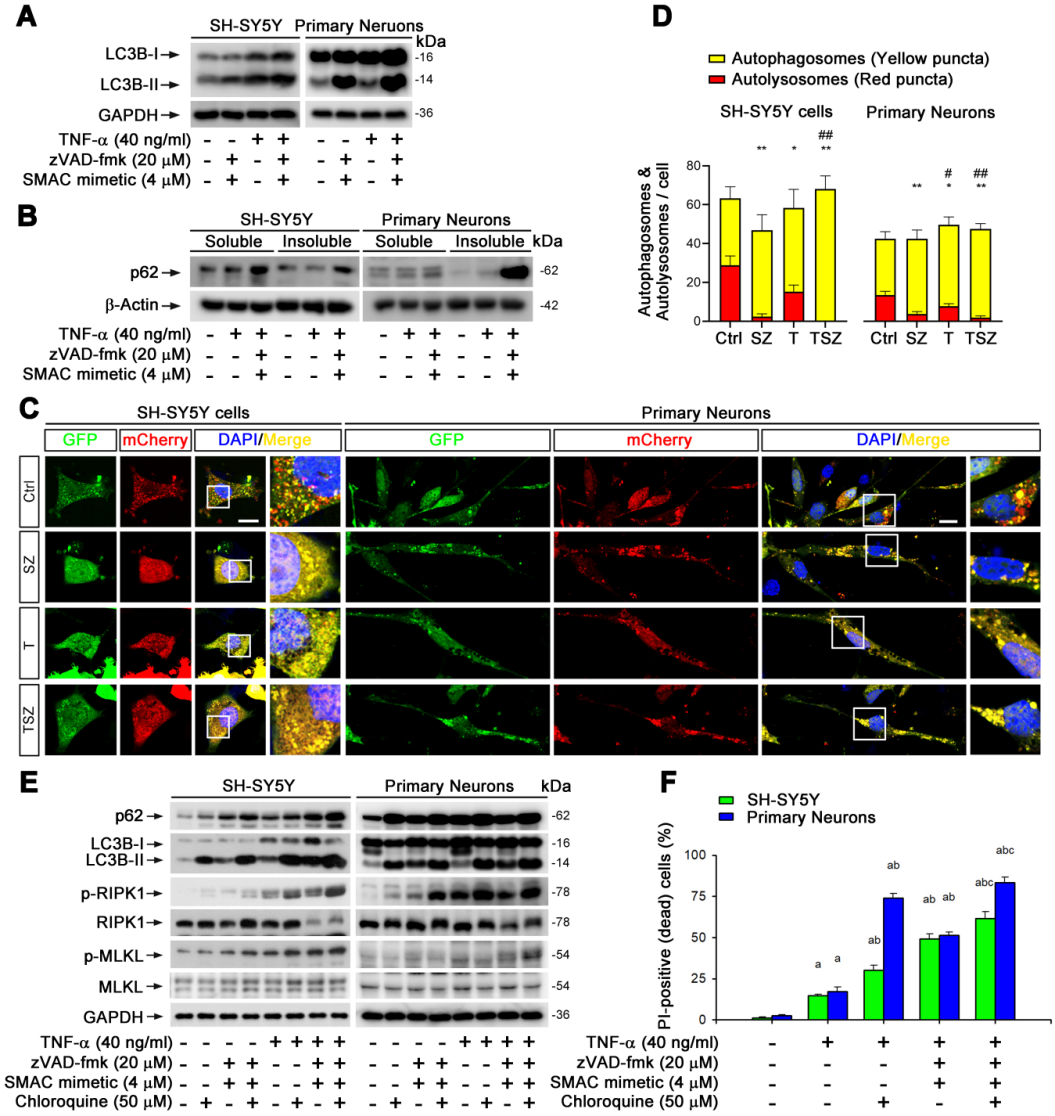
** Impaired autophagic flux is involved in neuronal necroptosis. (A)** Immunoblotting shows the levels of LC3 with indicated each TSZ treatment in SH-SY5Y cells and primary neurons. **(B)** Immunoblotting of soluble and insoluble protein extracts from SH-SY5Y cells or primary neurons probed with an anti-p62 antibody. **(C)** Representative images of SH-SY5Y cells or primary neurons transduced with adenovirus expressing GFP-mCherry-LC3. Cells without TSZ treatment as control (Ctrl). Total autophagosomes (yellow puncta) and functional autophagolysosomes (red only puncta) are visualized and images in white rectangles are amplified. **(D)** Quantitation of autophagosomes (yellow puncta) and functional autophagolysosomes (red only puncta) from fluorescence-labeled cells in (C). **(E)** Immunoblotting of cell lysates from the SH-SY5Y cells or primary neurons with indicated TSZ and/or Chloroquine treatments. **(F)** Quantitation of PI-positive cells from SH-SY5Y and neuronal cell cultures with indicated treatments. Scale bar, 5 µm. Error bar, mean ± SEM from at least 15 GFP/mCherry labeled cells; n=3~5 independent experiments; ^*^p < 0.05, ^**^p < 0.01, difference with control group in red puncta; ^#^*p* < 0.05, ^##^*p* < 0.01, difference with control group in yellow puncta; ^a^*p* < 0.05, difference with control group; ^b^*p* < 0.05, difference with TNF-α treated group; ^c^*p* < 0.05, difference with TSZ treated group; analysis was performed by one-way ANOVA test.

p62 is a critical molecule participating in several processes of autophagic flux including the initiation/assembly of autophagosome, docking/fusion of autophagosomes with lysosomes, and cargo degradation 33. We asked whether p62 upregulation during neuronal necroptosis is caused by impaired autophagic flux. We checked the state of autophagic flux by monitoring the protein levels and cellular localization of LC3, a key marker of autophagy. In all three cell culture models mentioned above, TNF-α treatment upregulated LC3-II, a standard indicator for autophagosomes, in a dose-dependent manner (Figure S7A). Strikingly, the levels of LC3-II and p62 were much higher with TSZ compared to TNF-α treatment (Figure 4B, 5A). Moreover, p62 levels in the insoluble fraction was sharply increased in TSZ treated cells (Figure 5B), suggesting that the formation of p62 aggregates is correlated with the inhibition of autophagy-mediated protein degradation.

To confirm this notion, we used recombinant adenoviral particles encoding GFP and mCherry tagged LC3 (Ad-GFP&mCherry-LC3) to evaluate autophagic flux 33. As shown in Figure S7B & C, TNF-α gradually reduced the number of red dots (mCherry without GFP fluorescence puncta, representing autolysosomes) and increased the number of yellow dots (co-localization of mCherry with GFP fluorescence puncta, representing autophagosomes). This change was notably further amplified by treatment with zVAD and Smac mimetic (Figure 5C & D), suggesting that TNF-α can impair neuronal autophagic flux. To clarify whether the impaired autophagic flux contributes to necroptosis, we used an autophagic flux inhibitor chloroquine (CQ) that can effectively raise the lysosomal pH and inhibit proteases 33. Of note, CQ sharply increased the upregulation of p62 and LC3-II triggered by TNF-α or TSZ treatment and enhanced the activation of RIPK1 and MLKL, as well as the consequent neuronal necrosis (Figure 5E & F).

Our data thus clearly show that impaired neuronal autophagic flux contributes to TNF-α-induced neuronal necroptosis.

### UVRAG down-regulation by TSZ leads to neuronal necroptosis

**Figure 6:**
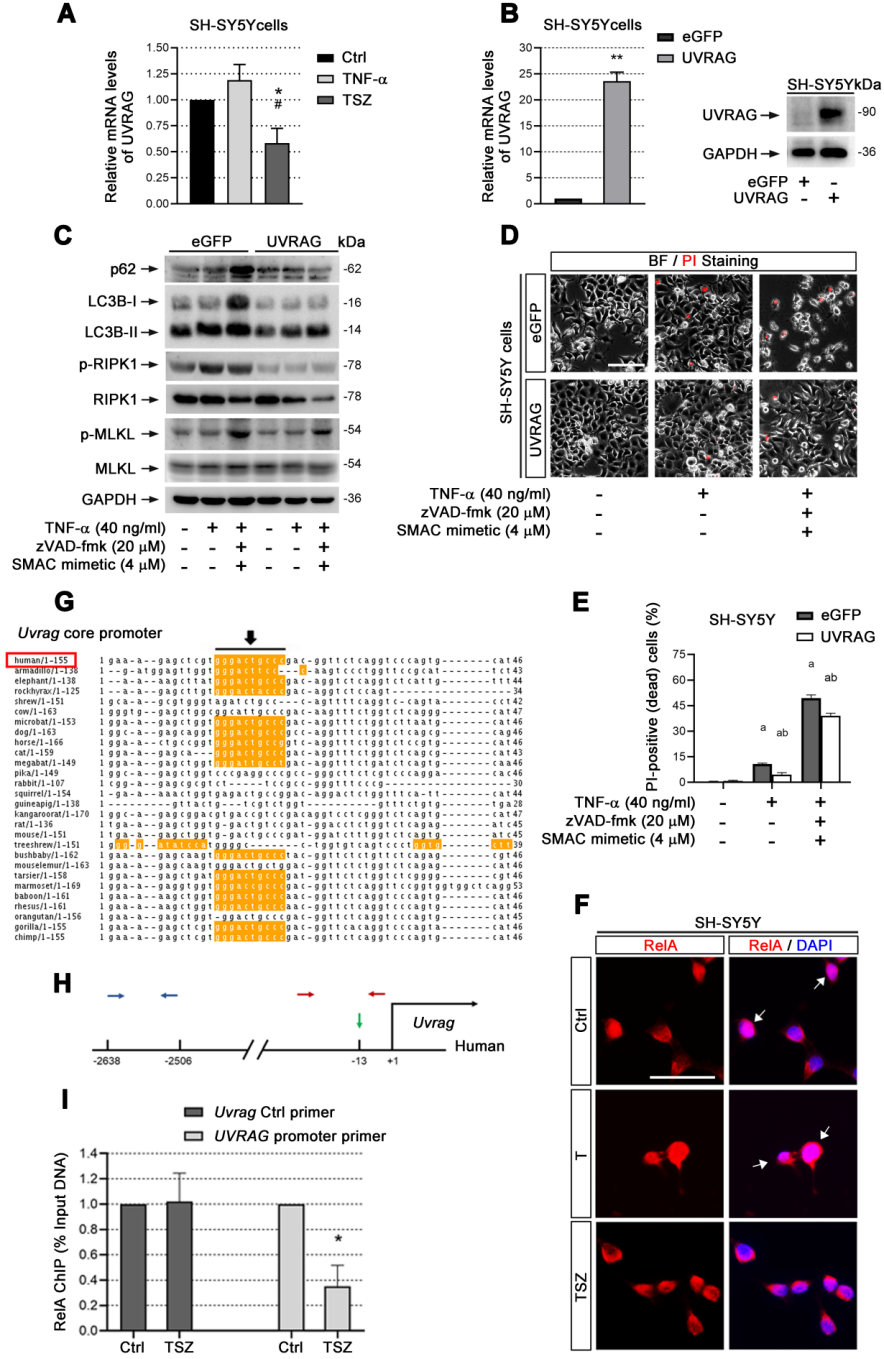
** UVRAG downregulation promotes cell necroptosis. (A)** qPCR assays show the UVRAG mRNA levels in SH-SY5Y cells with indicated treatments. The mRNA levels in Ctrl groups (without treatment) were normalized as 1. **(B)** qPCR and immunoblotting assays show the levels of UVRAG mRNA and protein levels in SH-SY5Y cells transduced with lentiviral particles expressing eGFP (Ctrl) or UVRAG. The mRNA levels in Ctrl groups were normalized as 1. **(C)** Immunoblotting of cell lysates from SH-SY5Y cells expressing eGFP or UVRAG with indicated treatments. **(D)** Representative images of PI staining in SH-SY5Y cells expressing eGFP (upper) and UVRAG (lower) after indicated treatments. **(E)** Quantitation of PI-positive cells from SH-SY5Y cell cultures with treatments as in (D). **(F)** IF images show the nuclear localization of RelA (red) in SH-SY5Y cells before or after T/TSZ treatments. Nuclei were counterstained with DAPI (blue). **(G)** Sequence alignment of NF-κB binding sites in the core promoter of UVRAG across species. Yellow marks predicted high-scoring NF-κB binding sites. Black arrows indicate the positions of NF-κB binding sites. Red rectangle, human. **(H)** Schematic diagram of UVRAG promoter in human. Green arrow indicates the position of NF-κB binding site. Red arrows indicate the positions of promoter primers designed for chromatin IP (ChIP)-qPCR. Blue arrows indicate the position of control primers for ChIP-qPCR. **(I)** ChIP-qPCR assays show UVRAG promoter binding activity of RelA in SH-SY5Y cells with or without TSZ treatment. The DNA levels in Ctrl groups were normalized as 1. Scale bar, 25 µm.^ *^p or ^a^*p* < 0.05, difference with Ctrl group; ^**^p < 0.01, difference with Ctrl group; ^#^*p* or ^b^*p* < 0.05, difference with eGFP group. Data represent mean ± SEM, n=3~5 independent experiments; analysis was performed by one-way ANOVA test.

We extended our study to the molecular mechanism of autophagy in TNF-α-mediated neuronal necroptosis. We examined mRNA expression levels of a series of genes responsible for the recognition and fusion of autophagosome and lysosome 34. Real-time quantitative PCR (RT-qPCR) data showed that among the tested genes, UVRAG mRNA, was the most significantly downregulated by TSZ treatment (Figure 6A, Figure S7D), suggesting that TSZ may disrupt neuronal autophagic flux by down-regulating UVRAG. To test this possibility, we constructed a SH-SY5Y cell line stably expressing UVRAG. Data showed that UVRAG overexpression reversed TSZ-induced autophagic flux impairment and necroptosis activation by preventing LC3-II and p62 upregulation as well as RIPK1/MLKL phosphorylation (Figure 6B-E). Hence, our data infer that down-regulation of UVRAG transcription by TSZ impairs neuronal autophagic flux, leading to p62 accumulation and the onset of necroptosis.

### UVRAG transcription is reduced through inactivation of NF-κB

cIAP1/2 facilitates TNF-α-induced transcription factor NF-κB activation 35. Given that Smac mimetic(S), as an inhibitor of cIAP1/2, can induce strong neuronal necroptosis (Figure 2), we speculated that TSZ-induced UVRAG downregulation resulted from NF-κB inhibition. Therefore, we checked the level of RelA (p65), a key subunit of NF-κBs, before and after TSZ treatment. Compared to TNF-α alone, TSZ did not alter the levels of RelA in cytoplasm, but reduced the levels in nuclei, indicative of an inhibition of NF-κB activation (Figure 6F).

Next we analyzed the core promoter regions of *Uvrag* gene across species using ConTra v2 software and found a high-scoring NF-κB binding site (Figure 6G & H). Chromatin-IP (ChIP) qPCR showed that NF-κB indeed bound to the indicated region of the *Uvrag* promoter (Figure 6I). Importantly, TSZ significantly reduced the binding of RelA to the *Uvrag* promoter (Figure 6I). We thus conclude that *Uvrag* is a direct target of TNF-α/TNFR1/NF-κB signaling and TSZ reduces UVRAG transcription by inhibiting NF-κB action.

### Upregulation of UVRAG reduced neuronal necroptosis in AD

**Figure 7:**
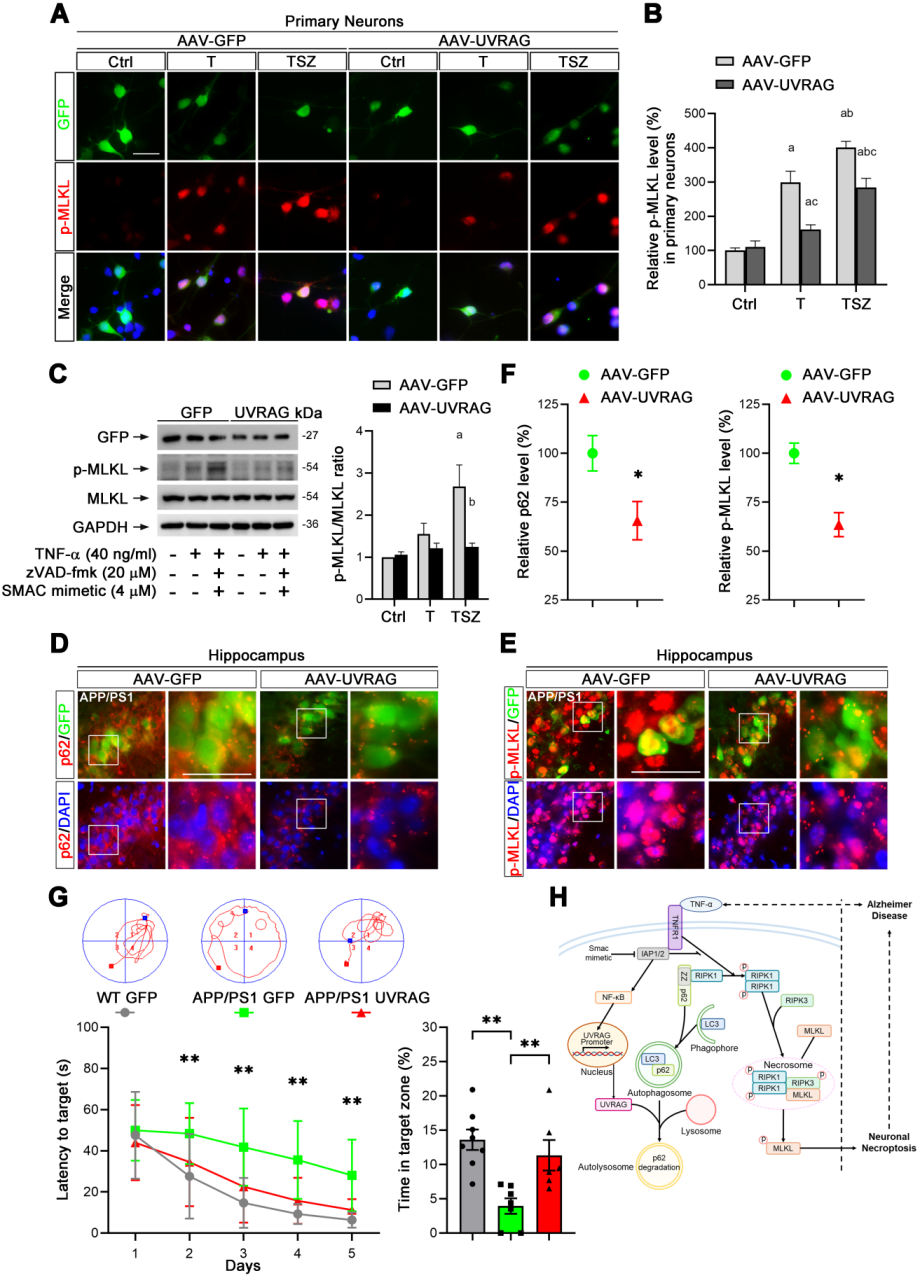
** UVRAG overexpression inhibits cell necroptosis in neuronal cultures and AD mice. (A)** IF images show p-MLKL levels (red) in primary neurons transduced with AAV particles expressing GFP (as control) or UVRAG after indicated T or TSZ treatments. **(B)** Quantitation of p-MLKL levels from GFP-labeled (green) neurons in (A). The p-MLKL levels in Ctrl groups were normalized as 100%. **(C)** Immunoblotting of cell lysates from primary neurons transduced with AAV-GFP or AAV-UVRAG with indicated T or TSZ treatments (left). The histogram (right) shows the ratio of p-MLKL to MLKL levels calculated from the blots. **(D)** IF images show the levels of p62 (red) in APP/PS1 injected with AAV-GFP (left panels) or AAV-UVRAG-GFP (right panels). **(E)** IF images show the levels of p-MLKL (red) in APP/PS1 injected with AAV-GFP (left panels) or AAV-UVRAG-GFP (right panels). **(F)** Quantitation of p62 and p-MLKL levels from GFP-labeled cells (green) in (D) and (E). **(G)** The quantitation shows the escape latency in Morris water maze (MWM) in the acquisition trials (left). The representative trajectories of MWM from each group are shown at top. The right histogram shows the time spent in the target quadrant in the probe trials. WT group (injected with AAV-GFP, n=8); APP/PS1 group (injected with AAV-GFP, n=7); APP/PS1 group (injected with AAV-GFP, n=6). **(H)** A graphical summary of the present study. Scale bar, 25 µm. Error bars indicate mean ± SEM from at least 20 RFP-labeled cells, n=3 mice per group or 3 independent cell cultures.

Based on our findings, we considered that UVRAG upregulation may inhibit neuronal necroptosis in AD mice. To test this hypothesis, we utilized AAV-mediated overexpression of UVRAG (Figure S4A) in primary neuronal cultures. UVRAG overexpression did markedly suppress TNF-α-induced MLKL phosphorylation (Figure 7A & B), which was further confirmed by immunoblotting in primary neurons (Figure 7C). We next transduced cells in hippocampal CA1 regions with AAV expressing UVRAG or control GFP in APP/PS1 mice at 10-month age by intrahippocampal injection. After 3 weeks, we sacrificed the APP/PS1 mice and measured p62 and p-MLKL levels in transfected neurons. IHC images showed the average level of p62 in UVRAG-overexpressing neurons was 34.5% lower than that in control GFP expressing neurons (Figure 7D, F left). Notably, UVRAG overexpression also lowered MLKL phosphorylation by 36.5% (Figure 7E, F right). In addition, UVRAG overexpression apparently alleviated learning and memory deficits of APP/PS1 mice, as assessed by the Morris water maze (Figure 7G). These results support the notion that upregulation of UVRAG inhibits neuronal necroptosis and improves cognitive function in AD mice, and that UVRAG may thus be a potential target for AD intervention.

## Discussion

Brain deterioration caused by neuronal loss is the most consequential feature of AD pathogenesis 36. The mechanistic study of neuronal loss is an object of even more intense study because neuronal loss starts at the early stage of AD, when Aβ plaques and neurofibrillary tangles have not yet appeared 37, 38. Therefore, targeting neuronal loss might slow or even prevent the progression of AD. According to the amyloid cascade hypothesis, Aβ accumulation serves as an initial signal in AD, which leads to neurofibrillary tangles and facilitates neuronal death and cognitive defects 39. A huge body of evidence shows that different forms of Aβ, either insoluble aggregates or soluble oligomers, can cause neuronal death by a rapid toxic effect on neurons 6 or by triggering chronic neuroinflammation and toxic cytokine release 7.

As for the mechanism of neuronal cell death, distinct processes including apoptosis, necrosis, autophagy, and parthanatos have been described in AD as well as other NDs 40. Necroptosis, a programmed cell necrosis, is a more recently discovered pathway to cell death detected in several NDs 41-44. In AD, neuronal necroptosis activation has been observed in the brains of AD patients and rodent AD models 11, 45. Recently, decreased O-linked β-N-acetylglucosaminylation in AD has been inferred to be a factor for promoting necroptosis 46. Notably, necroptosis detected in granulovacuolar degeneration (GVD) is associated with neuronal loss in AD, which correlates well with Tau pathology and abnormal aggregation of RNA binding proteins and ER chaperones in GVD 47. Thus, necrosome detected in GVD may represent an AD-specific form of necroptosis-related neuronal death 45. In line with these findings, our data also find upregulated protein levels of MLKL or p-MLKL, a necroptotic marker, in the brains of AD patients, APP/PS1 and 5xFAD mice (Figure 1A-D).

Various extracellular stimuli can activate necroptosis, including TNF, interferon, and Toll-like receptor (TLR) signaling as well as viral infection 48. But the question has then been raised, how is neuronal necroptosis in AD initiated? Given the accumulated evidence of elevated TNF-α in the CSF and brains of AD patients 20, 49, we hypothesized that upregulated TNF-α in AD brain might lead to the activation of neuronal necroptosis. Using injection of recombinant TNF-α, an anti-TNFR1 neutralizing antibody, and AAV particles expressing TNFR1 shRNA, we provide evidence that activated neuronal necroptosis in APP/PS1 hippocampus is dependent on TNF-α/TNFR1 signaling (Figure 1F-J). In addition, using three different types of neuronal cell culture models, we also successfully observed that TNF-α treatment could induce cell necroptosis *in vitro* (Figure 2). Our data also support the notion that neuronal necroptosis is likely a secondary source of cell death induced by Aβ-mediated neuroinflammation at a later stage in AD, consistent with previous findings that necroptosis inhibitor Nec-1 can block Aβ-induced cell death in APP/PS1 mice, but with limited efficacy at early stages 50.

Turning to details of the inferred pathway, Figure 7H outlines the cascade of interacting molecules and processes involved in the initiation and level of necroptosis. In the overall fate of neurons, RIPK1 is a key regulator of survival signaling, inflammation response, and pro-death stimuli in many human diseases such as autoimmune and NDs 51. TNF-α/TNFR1 mediated RIPK1 activation is the most comprehensively studied signaling pathway; it decides whether cells live or die by tightly regulating pro-survival NF-κB action, pro-apoptotic caspase activity, and necroptotic MLKL activation 52. We used our neuronal culture models and confirmed that RIPK1/RIPK3/MLKL cascade was activated upon TNF-α or TZS stimulation (Figure 3A). Consistently, RIPK1 inhibitor Nec-1 and RIPK1 KD both prevented phosphorylation of RIPK1/RIPK3/MLKL cascade (Figure 3A-G). We also saw the increased oligomerization of RIPK1 in TNF-α-induced necroptosis (Figure 3B), possibly due to the inhibition of cIAP1/2 by Smac mimetic and blocking of ubiquitylation at the K377 site targeting RIPK1 degradation 23, 35.

To further explore the mechanism of RIPK1-dependent neuronal necroptosis in AD, we focused on p62/SQSTM1, a RIPK1 binding partner that participates in both the ubiquitin-proteasome system (UPS) and autophagic lysosomal degradation process in many diseases 53. In our cell culture models, we verified the complex formation of p62, RIPK1, RIPK3 and MLKL (Figure 4A). Some literature has reported lower p62 expression in the brains of AD patients and rodent models of AD 54, 55; but one group reported increased p62 levels in the hippocampus of AD patients 56. This discrepancy may result from biological differences among patients or in different animal models. In our own study, data consistently show higher levels of p62 in TNF-α-treated cells in cultures, hippocampal CA1 cells in APP/PS1 mice, and neuronal cells in the cortexes of AD patients (Figure 4B & C; 4F & G). We further find that p62 KD suppresses the activation of neuronal necroptosis in both cell cultures and in APP/PS1 mice (Figure 4D & E; 4H & I). Our results thus imply that p62 inhibition, a therapeutic modality in prostate cancer 57, could be a possible candidate for AD intervention.

p62 is also implicated in the interacting pathway of autophagy. Impaired autophagic flux has been found to be able to induce cell death by disabling degradation of protein aggregates in NDs 17, 58. p62 was accumulated in both soluble form and in insoluble aggregates after TNF-α treatment (Figure 5B). Notably, autophagic flux was seen to be inhibited, also mediating RIPK1-dependent necroptosis (Figure 5, Figure S7A-C). TNF-α can damage autophagic flux by disrupting lysosome acidification and blocking the degradative function of autolysosomes 59. These hints led us to search for possible molecules involved in regulating autophagic flux. We examined the mRNA levels of major genes participating in autophagosome recognition and autolysosome fusion. Among the tested targets, UVRAG mRNA was the most one strikingly reduced in level by TSZ treatment (Figure 6A, Figure S7D). UVRAG is in fact a critical modulator of several steps in autophagic flux, including autophagosome-lysosome fusion 34, 60. In agreement with these findings, our data clearly show that UVRAG overexpression can alleviate the impairment of autophagic flux and reduce RIPK1-dependent necroptosis (Figure 6B-E). UVRAG downregulation can likely disrupt autophagosome-lysosome fusion, resulting in p62 accumulation, which then further recruits RIPK1 to autophagosome and facilitates its oligomerization and auto-phosphorylation (Figure 7H). Consistently, AAV-mediated UVRAG overexpression reduced TNF-α-induced p-MLKL upregulation in both neuronal cultures and in APP/PS1 mice (Figure 7A-F). Strikingly, UVRAG overexpression in AD mice even improved cognitive function (Figure 7G). These findings thus provide evidence that upregulation of UVRAG may be another route to suppress neuronal necroptosis in AD.

Overall, in the picture of TNF-α/TNFR1-dependent neuronal necroptosis in AD, there appear to be several key successive events, including UVRAG downregulation and autophagic flux impairment and p62 accumulation, which further leads to RIPK1 activation and subsequent neuronal necroptosis (Figure 7H). It remains unknown how other molecules and signaling pathways are modulated to precisely set levels of cell necroptosis in AD. However, in a broader view, our results have demonstrated that inflammatory TNF-α signaling, pro-survival NF-κB action, autophagic machinery, and RIPK1 activity are all tightly controlled and unbalanced during neuronal necroptosis in AD. This may provide a general outline of the mechanism of neuronal necroptosis in other NDs as well.

## Materials and Methods

### Human brain tissues

Paraffin sections of the human cerebral cortex were kindly provided by NIH NeuroBioBank. These materials include samples from 8 AD patients (Braak VI) and 7 age-matched healthy elderly.

### Mice

APP/PS1 (JAX, Stock # 034848) and 5×FAD (JAX, Stock # 034848) transgenic mice were purchased from The Jackson Laboratory and generously provided by Dr. Guobing Chen (Jinan University, China). In this study, we crossed the APP/PS1 or 5×FAD with C57BL/6 mice (GemPharmatech, China) to obtain daughter mice for further study. All mice were maintained under standard laboratory conditions with free access to food and water unless otherwise indicated. All experiments were conducted in accordance with the regulations for the Administration of Affairs Concerning Experimental Animals (China) and approved by the China Pharmaceutical University Animal Ethics Committee. Mice were genotyped by One Step Mouse Genotyping Kit (Vazyme, Cat# PD104-01), according to manufacturer's instructions.

### Immunohistochemistry (IHC), immunofluorescence (IF) and autophagic flux analysis

For DAB staining of paraffin sections, 5 µm slices were fixed in 10% formaldehyde followed a protocol as described 61. For Immunofluorescence staining of cryostat sections, 30 µm frozen slices were sectioned by a Leica CM1950 microtome and followed a protocol as previously described 61. All antibodies used in this study were listed (Table S1). Images were analyzed by Olympus IX73 microscopy.

Images of cellular autophagic flux analysis were captured by the Olympus FV1000 laser scanning confocal microscope. For quantitative analysis of the fluorescence intensity, the integral optical density was measured by using Image-Pro Plus 6.0 software.

### Brain stereotaxic injection

Brain stereotaxic injection was performed as described 62, 63 with modification. In brief, for lateral ventricle injection of TNF-α, 2 μL recombinant murine TNF-α (5 μg in 2 μl of PBS) or 2 μL PBS (Ctrl) was injected into the lateral ventricle at -0.4 mm from bregma, mediolateral 1.0 mm, depth 2.5 mm of mouse brains using a digital pump (ZS Dichuang Company, China) at a speed of 0.25 μl/min with a microinjection syringe. After injection, the syringe was remained in the injection point for additional 5 min before it was slowly removed. For hippocampal CA1 injection, same instruments and procedure applied, except 4 μL TNFR1 antibody (Thermo Fisher Scientific, Cat# 16-1202-85) or 2 μL viral particles (10^12^~10^13^ v.g./ml) was injected into the hippocampal CA1 region at -2.2 mm from bregma, mediolateral 1.7 mm, depth 2.4 mm of mouse brains. All mice were placed on a heating pad until they recovered from the surgery. One week after injection of TNF-α (or) TNFR1 antibody, while three weeks after virus injection, mice were sacrificed for further experiments.

### Cell culture

Human SH-SY5Y cells were cultured in DMEM with 10% FBS. Rat PC12 cells were cultured in DMEM with 5% FBS (Gibco) plus 10% horse serum (Hyclone). The primary mouse cortical neuron culture was performed as described 64, In brief, cortical neurons were isolated from E16-E18 mouse brains and cultured in Neurobasal (Gibco, Ca# 21103049) medium supplemented with B-27 (Gibco, Ca# 17504044) on Poly-D-Lysine (Sigma Aldrich, Ca# P7405) coated 6-well or 96-well plates. Cells were maintained at 37 °C in an incubator containing 5% CO_2_. The cells were pretreated with or without zVAD-fmk (MCE, Ca# HY-16658B), Necrostatin-1 (MCE, Ca# HY-15760) or Chloroquine (MCE, Ca# HY-17589) for 1 h followed by adding recombinant human or murine TNF-α (or PBS) (PeproTech, Cat# 300-01A or 315-01A) in combination with (or without) SMAC mimetic (MCE, Ca# HY-12600) for 8 h for protein extraction, RNA/DNA extraction, IHC and cellular autophagic flux analysis. In some cases, cells were cultured for 24 h for cell viability analysis, Propidium Iodide (PI) staining, and Acridine Orange (AO) staining.

### Preparation of adeno-associated virus (AAV), lentivirus and adenovirus

The AAV vector expressing two shRNA sequences and a separated RFP (pAV-2in1shRNA-CMV-RFP) was obtained from Vigene Biosciences, Inc. The structure of AAV vector can be found in Supplementary information (Figure S4). The below is shRNA sequences. TNFR1 shRNA-1: 5'-CCTCGTGCTTTCCAAGATGA-3'; TNFR1 shRNA-2: 5'-CCCGAAGTCTACTCCATCAT-3'; p62 shRNA-1: 5'-TAGTACAACTGCTAGTTATT-3', p62 shRNA-2: 5'-GAGGTTGACATTGATGTGGA-3'. The AAV vector expressing GFP (pAV-CAG-P2A-GFP) was obtained from Vigene Biosciences, Inc. HA-tag fused UVRAG was cloned into an AAV vector named pAV-CAG-P2A-GFP (Vigene Biosciences) (Figure S4). The viral particles (10^12^~10^13^ v.g./ml) were packaged and purified by Vigene Biosciences for further use.

For lentivirus production, HEK293T cells were transfected with lentiviral vectors and packing plasmids using a Lentiviral Packaging Kit (Yeasen, Ca# 41102ES20). The structure of lentiviral vectors (Yeasen) used were listed in Supplementary information (Figure S4). The shRNA sequences were listed as below. RIPK1 shRNA: 5'-CGGAACAGATTCTGGTGTCT-3'; p62 shRNA: 5'-CCTCTGGGCATTGAAGTTGA-3'. shRNA-resistant wild type p62 (p62-wt), zz domain deleted p62 (p62-zz△), and *Uvrag* genes were cloned into indicated vectors, respectively (Figure S4). SH-SY5Y cells were transduced with above lentiviral particle-containing supernatant in the presence of 8 mg/ml polybrene for 24 h, and then exposed to 2 mg/ml puromycin. The lentivirus encoding eGFP was used as a control.

The adenoviral particles expressing mCherry-GFP-LC3B protein (Ad-mCherry-GFP-LC3) and GFP-p62 fusion protein (Ad-GFP-p62) were purchased from Beyotime (Ca# C3011 and C3015). The control adenoviral particles expressing β-glactosidase (Ad-LacZ) were purchased from Sciben Biotech (Ca# AD1002). SH-SY5Y cells or primary neurons were transduced with the individual adenoviral particles for 24 h at 5 of multiplicity of infection (MOI=5). Ad-LacZ was used as a control.

### Cell viability analysis

Cells were seeded in 96-well plates before indicated treatment. The cells were then loaded with Enhanced Cell Counting Kit-8 (CCK-8) (Beyotime, Ca# C0046). Subsequently, the cell viability was determined by measuring the absorbance at 460 nm.

### Propidium iodide (PI) staining and Acridine orange (AO) staining

Cells were seeded in 6-well plates before indicated treatment. Then cells were incubated with 2 μg/ml propidium iodide (PI) (Sigma Aldrich, Ca# P4170) for 10 min or 200 μM Acridine orange (AO) (Sigma Aldrich, Ca# A6014) for 15 min as described 29. All PI stained wells were rinsed three times with PBS, all AO stained wells were washed with PBS three times before 4% FPA fixation for 20 min, followed by direct image capture using Olympus IX73 microscope. Primary neurons were stained with an anti-Tuj1 antibody.

### Immunoblotting, immunoprecipitation and insoluble protein extraction

Immunoblotting and immunoprecipitation were performed as described 65. For soluble and insoluble protein extraction, cells were lysed in cold PBS/1% Triton X-100 buffer (PBS containing 1% Triton X-100 and cocktail protease inhibitor) for 30 min on ice. After centrifugation at 15,000× g for 30 min at 4 °C, the supernatants were collected as soluble protein fractions. Pellets were washed with cold PBS/1% Triton X-100 buffer, and then re-suspended in 8 M urea containing 1% SDS, 1% Triton X-100 and cocktail protease inhibitor. After sonicated, the samples were centrifuged at 15,000× g for 15 min at 4 °C and the supernatants were collected as insoluble fractions. All the antibodies used were listed (Table S1).

### Quantitative real-time PCR (qPCR)

Cells were homogenized in Trizol reagent (Vazyme, Ca# R701-01) for extraction of total RNA based on the manufacturer's protocol. Purified RNA was reverse transcribed to cDNA using the HiScript III 1st Strand cDNA Synthesis Kit (Vazyme, Ca# R312-01). mRNA levels were determined by qPCR using AceQ qPCR SYBR Green Master Mix kit (Vazyme, Ca# Q131-02). All primers used are listed in Table S2.

### Core promoter analysis and chromatin immunoprecipitation

Core promoter analysis of *Uvrag* gene was performed using a computational program ConTra v2 (http://bioit.dmbr.ugent.be/contrav2/index.php). Chromatin was extracted and immunoprecipitated using a Chromatin-IP (ChIP) kit (CST, #9004) and an anti-RelA antibody (CST, #8242) according to the manufacturer's protocol. Then separated and purified DNA was amplified by qPCR. All primers used are listed in Table S2.

### Morris Water maze

Morris water maze (MWM) test was performed as described 66. In brief, mice were trained to localize a hidden escape platform (10 cm diameter, 1cm below the surface of the water) in a circular pool (130 cm diameter, 60 cm deep) with water filled to 30 cm depth at about 22 °C. The escape platform was placed in the center of the south-west quadrant. The training consisted of 5 days with 4 trail per mice per day. The mice were released facing to the pool wall at different entry location (north, east, south-east and north-west), in an order that varied from day to day. Swimming was automatically video-tracked until the subject found the hidden escape platform and remained at least 2 s on it, or until a maximum of 60 s. Mice that did not locate the escape platform over 60 s were guided to the hidden platform until they remained at least 10 s on it. After visible platform trials, escape platform was removed before mice were released facing to the pool wall at the north-east entry point. The probe trials were video-tracked for 30 s.

### Data and statistical analyses

All data were expressed as mean ± SEM. Student's *t*-test for nonpaired replicates was used to identify statistically significant differences between treatment means. Group variability and interaction were compared using either one-way or two-way ANOVA followed by Bonferroni's post-tests to compare replicate means. Significance was accepted at *p* < 0.05.

## Supplementary Material

Supplementary figures and tables.

## Abbreviations

AAV: Adeno-associated viral vectors
Aβ: Amyloid beta
AD: Alzheimer's disease
Ad: adenoviral
AO: Acridine Orange
Ctrl: Control
CQ: chloroquine
CSF: cerebrospinal fluid
IF: immunofluorescence
IHC: Immunohistochemistry
KD: knockdown
MCI: mild cognitive impairment
MLKL: Mixed Lineage Kinase Domain Like Pseudokinase
Nec-1: Necrostain-1
NDs: neurodegenerative diseases
PI: Propidium iodide
p62/SQSTM1: Sequestosome 1
RIPK1: Receptor Interacting Serine/Threonine Kinase 1
RIPK3: Receptor Interacting Serine/Threonine Kinase 3
S: Smac mimetic
sc: scrambled control
shRNA: small hairpin RNA
T: TNF-α
TNF-α: Tumor necrosis factor alpha
TNFR1: Tumor necrosis factor receptor 1
Tuj1: Neuronal class III β-tubulin
UPS: ubiquitin-proteasome system
UVRAG: UV Radiation Resistance Associated
zVAD/z: zVAD-fmk
