# Supplementary material for: TNF-α-dependent neuronal necroptosis regulated in Alzheimer's disease by coordination of RIPK1-p62 complex with autophagic UVRAG
Source: Theranostics. 2021 Sep 13;11(19):9452–69. doi: 10.7150/thno.62376 (PMC8490500; doi:10.7150/thno.62376)
Supplement: Supplementary file 1 — Supplementary figures and tables. [file thnov11p9452s1.pdf]

# Supplementary Materials

# TNF-α-dependent neuronal necroptosis regulated in Alzheimer's disease by coordination of RIPK1-p62 complex with autophagic UVRAG

Chong Xu[1#], Jialin Wu[1#], Yiqun Wu[1], Zhichu Ren[1], Yuyuan Yao[1], Guobing Chen[2], Libin Wei[3], Xijing Chen[4], Jian Sima[*1]

1. Laboratory of Aging Neuroscience and Neuropharmacology, School of Basic Medicine and Clinical Pharmacy, China Pharmaceutical University, Nanjing, 210009, China.
2. Institute of Geriatric Immunology, School of Medicine, Jinan University, Guangzhou, 510632, China.
3. Jiangsu Key Laboratory of Carcinogenesis and Intervention, China Pharmaceutical University, Nanjing, 210009, China.
4. Clinical Pharmacokinetics Laboratory, School of Basic Medicine and Clinical Pharmacy, China Pharmaceutical University, Nanjing, 211198, China.

# These authors contributed equally to this work.

* Correspondence to: simajian@cpu.edu.cn (J. S.)

**This PDF file includes:**

Supplementary Figure S1-7.

Supplementary Table S1-2.

## Supplementary Figures

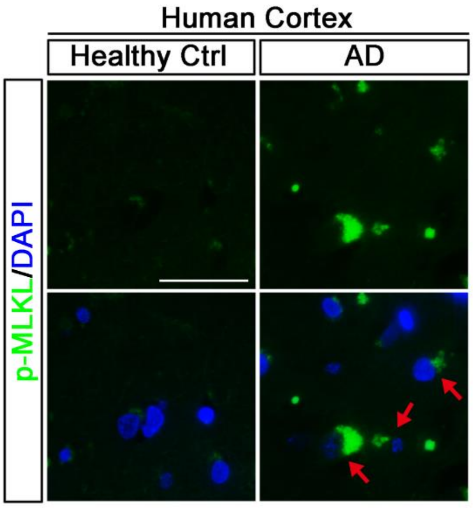


**Figure S1. Increased p-MLKL levels in AD brain**
IF images show the levels of p-MLKL (green) in the cortex from AD patients and healthy controls. Nuclei were counterstained with DAPI. Arrows indicate positive IF staining. Scale bar, 25 μm.

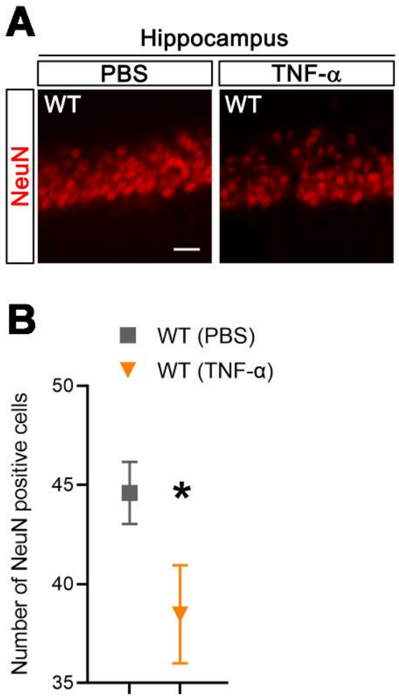


**Figure S2. TNF-α injection induces neuronal loss in hippocampal CA1 region**
(A) IF images show the number of neurons (red) in the in the hippocampal CA1 regions from WT mice (10-month old) with or without TNF-α injection. Neurons were stained with an anti-NeuN antibody. (B) Quantitation of the number from NeuN positive cells in (A). Scale bar, 25 µm.

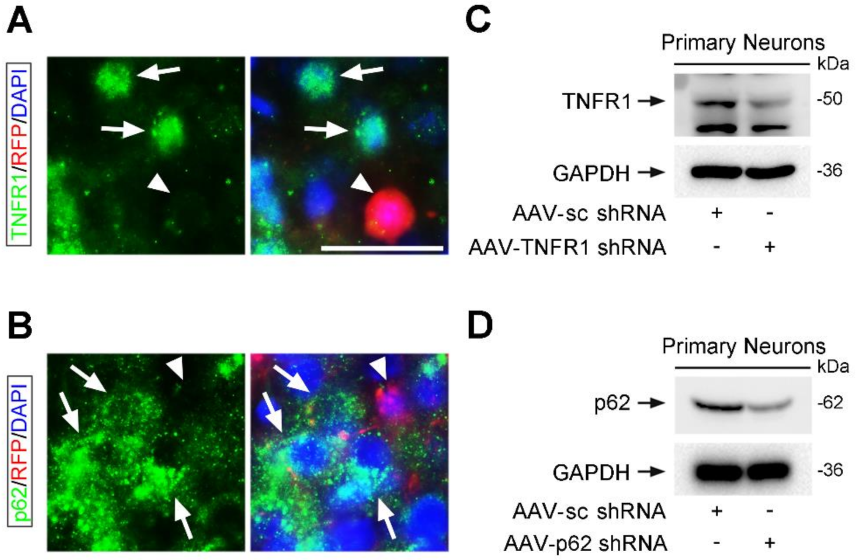


**Figure S3. The validation of AAV-mediated shRNA knockdown efficiency**
(A) IHC staining of TNFR1 (green) and RFP images (red) of hippocampal CA1 cells in APP/PS1 mice after injection with AAV particles. Nucleus were counterstained with DAPI. (B) As in (A), except IHC staining of p62 (green). Immunoblotting shows the levels of TNFR1 (C) or p62 (D) in primary neurons after transduction with AAV particles. GADPH as a loading control. Scale bar, 25 μm.

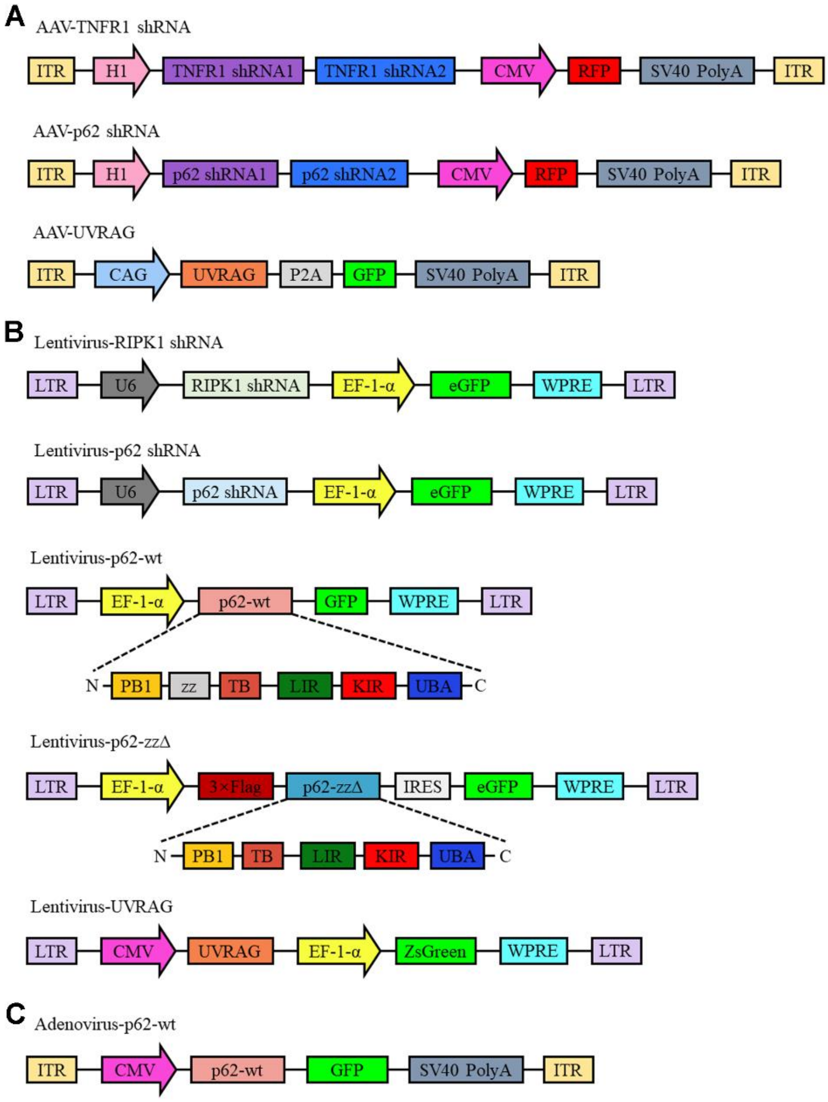


**Figure S4. Schematic presentation of the plasmid constructs.**
Schematic structures of the adeno-associated viral (AAV) plasmid constructs (A), HIV-1-based lentivial plasmid constructs (B), and an adenoviral plasmid construct (C) used in this study.

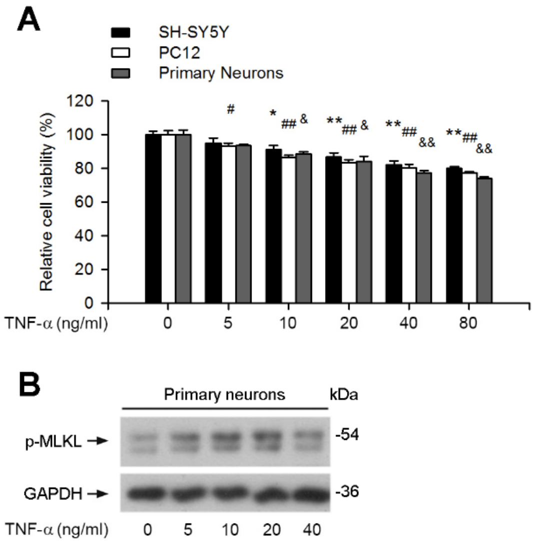


**Figure S5. TNF-α induces neuronal necroptosis.**

(A) Quantitation of cell viability from neuronal cell cultures after mTNF-α treatment with indicated concentrations. (B) Immunoblotting shows levels of p-MLKL in primary neurons after mTNF-α treatment with indicated concentrations. GADPH as a loading control. $^{*,\#,\&}p < 0.05$, difference with control group; $^{**,\#\#,\&\&}p < 0.01$, difference with control group. Data represent mean ± SEM, n=3-5 independent experiments; analysis was performed by one-way ANOVA test.

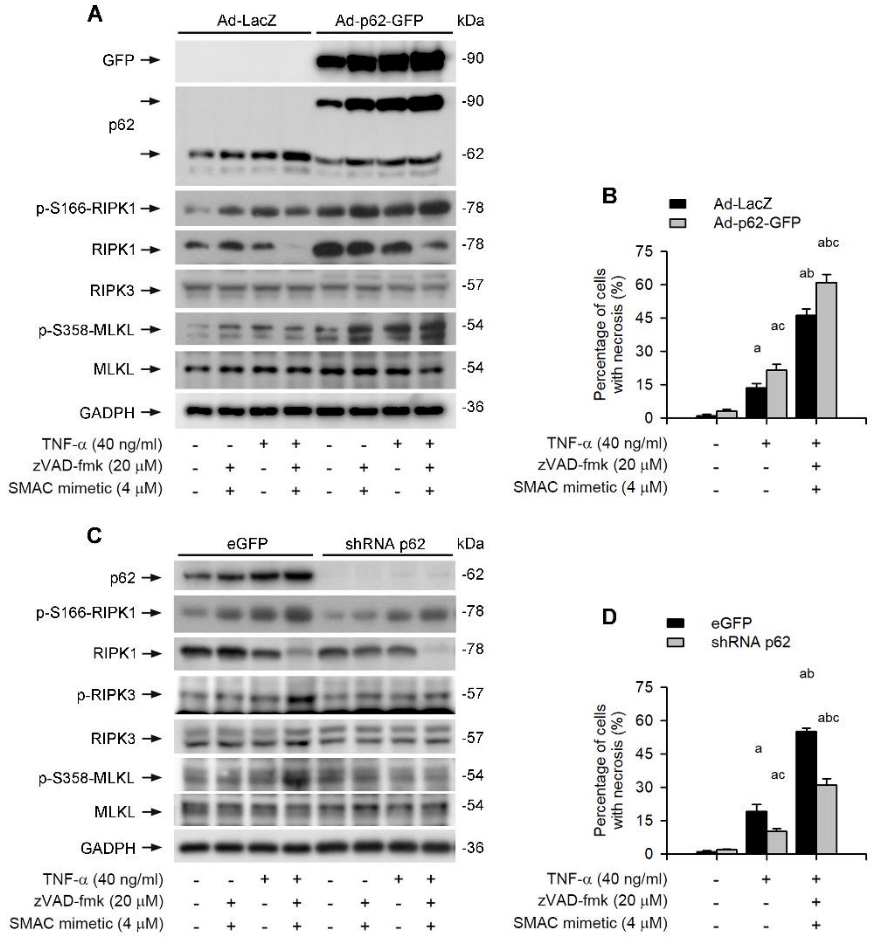


**Figure S6. p62 regulates TNF-α-induced neuronal necroptosis**

(A) Immunoblotting analysis of the SH-SY5Y cells transduced with adenoviral (Ad) particles encoding LacZ (as control) or p62-GFP with TSZ treatments using the indicated antibodies. GADPH as a loading control. (B) Quantitation of PI-positive cells from SH-SY5Y cells transduced with Ad-LacZ or Ad-p62-GFP after indicated treatments. (C) Immunoblotting analysis of the SH-SY5Y cells transduced with lentiviral particles encoding eGFP (as control) or p62 shRNA with TSZ treatments using the indicated antibodies. (D) Quantitation of PI-positive cells from SH-SY5Y cells transduced with lentiviral particles encoding eGFP or p62 shRNA after indicated treatments. $^{a}p < 0.05$, difference with control group; $^{b}p < 0.05$, difference with TNF-α treated group; $^{c}p < 0.05$, difference with LacZ or eGFP group. Data represent mean ± SEM, n=3-5 independent experiments; analysis was performed by one-way ANOVA test.

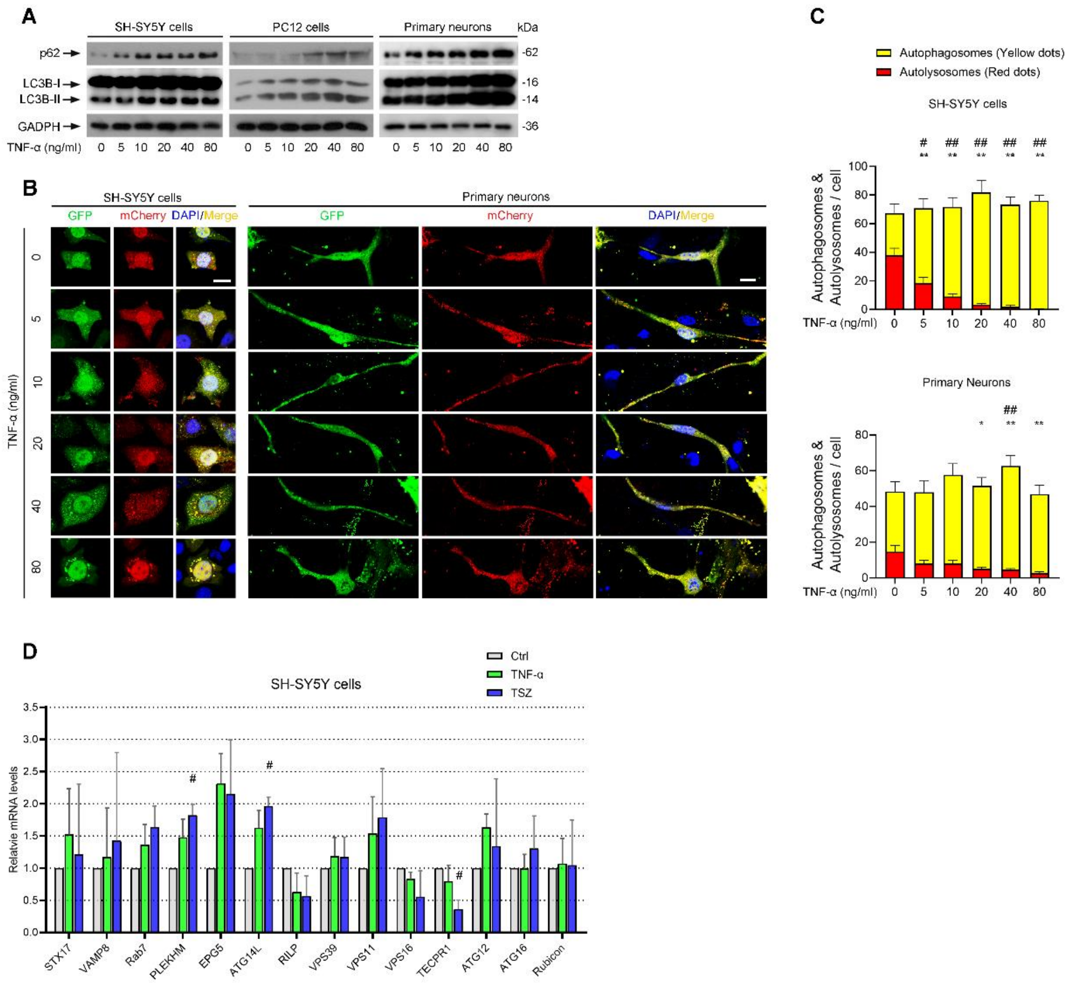


**Figure S7. Impaired autophagic flux is involved in TNF-α-induced neuronal necroptosis**

(A) Immunoblotting shows the levels of LC3 with different doses of TNF-α treatment in SH-SY5Y cells (left panels), in PC12 cells (middle panels) and in primary neurons (right panels). GADPH as a loading control. (B) Representative images of SH-SY5Y cells or primary neurons transduced with adenoviral particles encoding GFP-mCherry-LC3.Cells without TNF-α treatments as control (Ctrl). Total autophagosomes (yellow puncta) and functional autophagolysosomes (red only puncta) are shown. Scale bar, 5 μm. (C) Quantitation of autophagosomes (yellow puncta) and functional autophagolysosomes (red only puncta) from fluorescence-labeled cells in (B). (D) qPCR assays show the indicated mRNA levels in SH-SY5Y cells with TNF-α or TSZ treatments. The mRNA levels in control groups (without treatment) were normalized as 1. Error bars indicate mean ± SEM from at least 15 adenovirus-infected cells; n=3-5 independent in-vitro experiments; $^{*}p < 0.05$, $^{**}p < 0.01$, difference with control group in red puncta; $^{\#}p < 0.05$, $^{\#\#}p < 0.01$, difference with control group in yellow puncta; analysis was performed by one-way ANOVA test.

## Table S1. Antibodies used in this study

| Antibodies | Source | Identifier |
|---|---|---|
| anti-MLKL | Santa Cruz | Cat# sc-293201 |
| anti-MLKL | Abclonal | Cat# A19685 |
| anti-p-MLKL (S345) | Abcam | Cat# ab196436 |
| anti-p-MLKL (S358) | Abcam | Cat# ab187091 |
| anti-Aβ | Cell Signaling Technology | Cat# 2454 |
| anti-NeuN | Abcam | Cat# ab177487 |
| anti-TNFR1 | Thermo Fisher Scientific | Cat# 16-1202-85 |
| anti-TNFR1 | Proteintech | Cat# 21574-1-AP |
| anti-Tuj1 | Cell Signaling Technology | Cat# 5568 |
| anti-Caspase-8 | Abclonal | Cat# A0215 |
| anti-Caspase-3 | Abclonal | Cat# A0214 |
| anti-RIPK1 | Cell Signaling Technology | Cat# 3493 |
| anti-p-RIPK1 (S166) | Cell Signaling Technology | Cat# 65746 |
| anti-p-RIPK1 (S166) | Affinity | Cat# AF2398 |
| anti-RIPK3 | Abclonal | Cat# A5431 |
| anti-p-RIPK3 (T231/S232) | Abcam | Cat# ab205421 |
| anti-p-RIPK3 (S227) | Abcam | Cat# ab209384 |
| anti-p62 | Abclonal | Cat# A19700 |
| anti-p62 | Cell Signaling Technology | Cat# 39749 |
| anti-LC3B | Sigma | Cat# L7543 |
| anti-UVRAG | MBL | Cat# M160-3MS |
| anti-RelA | Cell Signaling Technology | Cat# 8242 |
| anti-GFP | Abclonal | Cat# AE012 |
| anti-Flag | Abclonal | Cat# AE063 |
| anti-GAPDH | Abclonal | Cat# AC002 |
| anti-Actin | Abclonal | Cat# AC004 |

## Table S2. RT-qPCR primers used in this study

| **Primer name** | **DNA sequence (5’-3’)** |
|---|---|
| UVRAG-qPCR F | CAGCAGATTCATGCCCGAAA |
| UVRAG-qPCR R | CTGGGCTCTATGAAGCCGTA |
| STX17-qPCR F | ATGTGTGTGGAGAGCGTCAA |
| STX17-qPCR R | ACAGTTCCACAAAGGCATCC |
| VAMP8-qPCR F | TGGGAGGAAGCCGACTAGGCG |
| VAMP8-qPCR R | ACTTTGCAGGTTCCGCACACGA |
| Rab7-qPCR F | ACAGACAAGTGGCCACAAAG |
| Rab7-qPCR R | CCGAGCAATTGTCTGGAAGG |
| TECPR1-qPCR F | CCCTTCAACGACCTCTCTGT |
| TECPR1-qPCR R | TCTCTGTACCACACCTTGCC |
| EPG5-qPCR F | CTGGACAGTGCTCAACATGG |
| EPG5-qPCR R | GGGCTTTCTTCAGAGCTTGG |
| ATG14L-qPCR F | ACCAGCATTAGCATCACG |
| ATG14L-qPCR R | AGGTCCTTGGGTTGTTTT |
| RILP-qPCR F | GGACACCAGAGGAAGCAGAG |
| RILP-qPCR R | GGATCAGGAGCTGGAGACTG |
| VPS39-qPCR F | TGAGAGACTTCCCAGAAGATG |
| VPS39-qPCR R | GGATGATGTGTTCCAGATAAGG |
| VPS11-qPCR F | CAAGCCTACAAACTACGGGTG |
| VPS11-qPCR R | GAGTGCAGAGTGGATTGCCA |
| VPS16-qPCR F | TACACGGCGAACTGGAACC |
| VPS16-qPCR R | GCCTCACACTAGCAGCTTTCT |
| TECPR1-qPCR F | CCCTTCAACGACCTCTCTGT |
| TECPR1-qPCR R | TCTCTGTACCACACCTTGCC |
| ATG12-qPCR F | TTTGCTAAAGGCTGTGGG |
| ATG12-qPCR R | AAGGAGCAAAGGACTGAT |
| Atg16-qPCR F | CACTAATATCTTTGGGAGACGCTCTG |
| Atg16-qPCR R | AACCGGGAACCTGGACTGAAC |
| Rubicon-qPCR F | AGTCCTTCTCCCACTGCTTC |
| Rubicon-qPCR R | TGAGATGGGCAGTGAGTCAG |
| UVRAG-ChIP F | TGCCTGGCACACCTTGTAGC |
| UVRAG-ChIP R | CTGAGAACCGTCGGGCAGTC |
| Ctrl-ChIP F | TGGCTCGGGAATAGCTAGTGT |
| Ctrl-ChIP R | GGGAGGAGGAAGTGGAGGCA |
